# Rehabilitation of Falls in Parkinson’s Disease: Self-Perception vs. Objective Measures of Fall Risk

**DOI:** 10.3390/brainsci11030320

**Published:** 2021-03-03

**Authors:** Kishoree Sangarapillai, Benjamin M. Norman, Quincy J. Almeida

**Affiliations:** Movement Disorders Research and Rehabilitation Centre, Wilfrid Laurier University, Waterloo, ON N2L 3J4, Canada; sang3490@mylaurier.ca (K.S.); bnorman@wlu.ca (B.M.N.)

**Keywords:** falls, gait, neurodegeneration, Parkinson’s disease, quality of life, rehabilitation, self -perception

## Abstract

Falls are an important cause of injury and increased hospital/long-term care facility stays and has been reported in 70% of people with Parkinson’s disease (PD), yet there is limited effectiveness of medications for reducing falls. As an adjunct, many exercise therapies succeed in objectively reducing the number of falls, but this may not translate to improved quality of life (QOL). Importantly, self-perceived fall risk has a greater influence on activities of daily living and QOL, making it important to evaluate in the rehabilitation of PD. The purpose of this study was to examine the influence of a 10-week exercise intervention (PD SAFE × TM) on self-perceived (according to balance confidence measures) and objective measures of gait that are commonly linked to fall risk in PD. Participants (*N* = 44) with PD completed PD SAFE × TM. Pre-/post-assessment involved the Activities-specific Balance Confidence Scale (perception), objective falls characteristics (stride time, stride width, stride length, and stride variability), and symptom severity (Unified Parkinson’s Disease Rating Scale motor subsection III (UPDRS-III)) after participants were stratified into a mild (no-balance impairment) vs. severe (balance impairment) groups. Overall disease severity (F (1, 43) = 8.75, *p* < 0.003) and all objective fall parameters improved (*p* < 0.05) in both groups, yet self-perceived fall risk improved in only the severe PD group F (1, 43) = 9.86, *p* < 0.022. Given that self-perceived fall risk and objective fall risk both play a role in the quality of life, identifying strategies to improve both aspects may be important in improving the overall quality of life.

## 1. Introduction

Parkinson’s disease (PD) is a progressive neurodegenerative disease that is characterized by several symptoms, including gait impairments and postural instability, which lead to falls [1]. Up to 70% of people with PD report a history of falling, which leads to not only physical injuries but also a fear of falling [1,2,3]. Together, this combination can greatly increase nursing home stays, and overall reduce a person’s quality of life (QOL) [3].

As falls can impact a variety of aspects of a person’s life, it is important to try to limit the detrimental effects of falls in people with PD. Unfortunately, the gold standard treatment for PD (dopaminergic medication) has a limited effect on falls [4]. While studies show medications may improve certain aspects of gait (stride length) [5,6], they have varying effects on postural instability and do not affect fall risk [4,5,6,7,8]. Due to the limitations of medications, exercise is often prescribed as an adjunct therapy to medication to manage the symptoms that medications do not improve. 

Exercise may be a valid adjunct therapy to medications as it can improve the gait impairments in PD. Recent studies have shown hydrotherapy, proprioceptive focal stimulation, Tai Chi, Agility Boot Camp, treadmill training have the ability to improve aspects of gait in PD [9,10,11,12,13,14]. However, improvements to fall-risk were not extensively studied. Several exercise programs have been shown to improve actual falls as well as gait characteristics that have been closely linked to falls (i.e., stride length, stride width, stride variability, stride time, and other spatiotemporal variables). However, actual falls are not the only factor contributing to fall risk [15,16,17,18,19,20,21,22]. Self-perceived fall risk, like symptoms of PD, worsens as the disease progresses, and plays an important role in overall fall risk, as it may dictate an individual’s activities of daily living (ADLs) and thereby QOL [23]. For example, if an individual perceives to be at high risk for falls, they may limit their ADLs. Limiting ADLs can cause immobilization and overall muscle weakness, leading to an increased risk of injury during ADLs and thereby poor overall QOL. In contrast, if an individual perceives to be at low risk for falls, they may put themselves in riskier situations where they may have a greater chance of falling. This may result in injuries, limiting their ADLs and leading to poorer QOL. Despite self-perceived fall risk playing a key role in the maintenance of ADLs, studies rarely examine the effects of an intervention on perceived fall risk. Therefore, the association between actual falls and self-perceived fall risk should be further explored by exercise studies aiming to improve overall fall risk. 

Of the many programs available for Parkinson’s, PD SAFE × TM is a sensory integration exercise program that consists of slow controlled movements where individuals are instructed to complete the exercises with and without vision. This intervention has been shown to improve overall gait and postural instability, increasing the likelihood of its ability to improve actual fall risk [24,25,26,27]. However, once again, actual fall risk is not indicative of overall fall risk. Thus, the purpose of this study was to examine the influence of a 10-week exercise intervention (PD SAFE × TM) on the gait characteristics that have been closely linked to falls and their relationship to self-perceived fall risk. Further, discrepancies in the relationship between self-perceived fall risk and actual falls between mild PD and severe PD were examined.

## 2. Materials and Methods

### 2.1. Participants

Participants with idiopathic Parkinson’s were recruited from the Movement Disorders Research and Rehabilitation Centre database at Wilfrid Laurier University, Waterloo, Canada. To be included, participants must understand verbal instructions in English and must not have a diagnosis of any other neurological disease other than PD. Prior to evaluation or participation, written informed consent was obtained according to the Declaration of Helsinki. The Research Ethics Board at Wilfrid Laurier University granted ethics approval (REB# 5801). This study has also been on clinicaltrials.gov (NCT03618901). A sample size calculation was conducted based on similar intervention studies in PD [16,28,29]. The power calculation was performed separately for self-perceived fall risk (using the Activities-Specific Balance Confidence Scale) and gait parameters linked to fall risk. In order to attain 80% power with an alpha level of 5%, a sample size of 44 was required to reach significance in self-perceived fall risk [29] and 50 subjects were required for gait parameters [16]. A total of 69 participants with idiopathic PD were screened from the MDRC database, of which 44 participants were recruited. Reasons for exclusion included unwillingness to participate and transportation difficulties. All individuals then took part in a 10-week PD SAFExTM intervention. Participants were then stratified into two groups based on Unified Parkinson’s Disease Rating Scale motor subsection III (UPDRS-III) disability score, mild (<20) (no balance impairment) and severe (>27) (more pronounced balance impairment) [30,31] (Table 1). 

### 2.2. Outcome Measures 

Primary Outcome Measures: Two primary outcome measures were used in this study, one was to measure self-perceived fall risk and the other was to measure gait characteristics that are closely linked to actual fall risk [3,7,32]. 

Self-perceived fall risk. An individual’s self-perceived fall was measured using the Activities-specific Balance Confidence scale (ABC) [33]. The ABC is a 16-item, highly reliable, and valid self-report measure in which participants rate their confidence that they will not fall while performing activities of daily living such as walking around the house [34,35,36]. For this assessment, a score of 0 indicates no confidence and a score of 100 represents complete confidence.

Actual fall risk. To measure actual fall risk, participants were asked to walk across the Zeno Walkway at a comfortable pace. Two walking conditions were assessed by a blinded assessor: 3 normal walking and 3 turns. During walking trials, participants were instructed to walk across a 9.75 m long and 0.61 m wide electronic walkway carpet (Zeno Walkway) at a comfortable pace. For the normal walking conditions, participants were instructed to start walking 2 m before the start of the carpet and continue walking 2 m after the end of the carpet to avoid collecting acceleration and deceleration data. For the turning condition, participants began walking 2 m before the start of the carpet, walked to the end of the carpet, turned 180 degrees on the carpet, and walked back the length of the carpet, continuing 2 m past the end of the walkway. 

These conditions were chosen as they most resembled day-to-day activities (i.e., walking around the house). Specifically, the mean stride time, stride length, stride width, and the variability of stride time, stride length, and stride width were analyzed. These gait parameters were chosen as previous research has described variability in stride length, time, and width to be closely linked to fall risk in the PD population [3,7,37]. Secondary Outcome Measure: To assess overall disease severity, the Unified Parkinson’s Disease Rating Scale (UPDRS-III) was evaluated by an experienced assessor.

### 2.3. Intervention

PD SAFEXTM is a 10-week progressive exercise intervention. PD SAFE × TM uses proprioceptive feedback to improve the motor symptoms of Parkinson’s. Materials used in the delivery of the intervention were chairs, Thera-bands, and a mirror. The exercise intervention consisted of a 30 min warm-up of non-aerobic walking exercise emphasizing body coordination. This was followed by 30 min of sensory attention focused exercise. This involved slow controlled movements. In order to enhance sensory feedback and proprioception, participants were instructed to complete the exercises first with their eyes open in front of a mirror and then with eyes closed. The intervention was delivered by a trained PD SAFE × TM instructor. The instructor was a Master’s student in Kinesiology, had two years of experience with PD SAFE × TM and Tri-Council Policy Statement: Ethical Conduct for Research Involving humans (TCPS-II) training. At least one trained volunteer was also present for each participant. Volunteers were senior Kinesiology students who had TCPS-II training and were trained in the delivery of PD SAFE × TM. The exercise was delivered in a group setting in front of a mirror. Sensory cues would then be given by the volunteers to fix positioning in both eyes open and closed conditions [24,25]. The exercise was delivered at the Movement Disorders Research and Rehabilitation at Wilfrid Laurier University. Participants took part in PD SAFE × TM for one hour, three times a week for ten weeks. All participants received that same exercise protocol, nothing was tailored or modified. Individuals were highly encouraged to attend every class. Both groups had high exercise adherence levels, mild (97.81%) severe (98.99%). The intervention was reported using the TIDieR guidelines.

### 2.4. Data Analysis

Data were analyzed using SPSS. Repeated measures ANOVA was conducted to determine overall improvement (from pre-assessment to post-assessment). Pearson correlations were conducted to determine the association between self-perceived fall risk and actual fall risk. An alpha level of 0.05 was set for significance.

## 3. Results

### Outcome Measures

**Figure 1:**
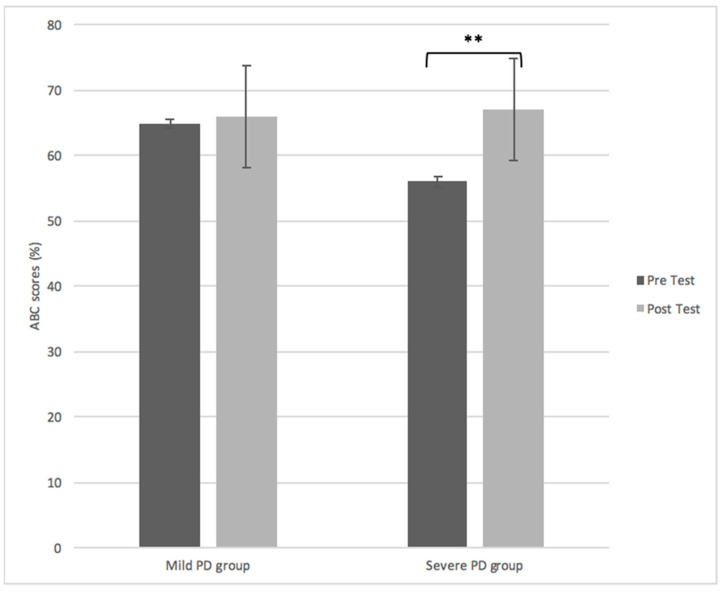
Activity-based confidence scores for the mild vs. severe PD group before and after PD SAFE × TM. Self-perceived fall risk significantly improved only for the severe group after PD SAFE × TM *p* < 0.022. The asterisk indicates significance *p* < 0.05.

A repeated measures ANOVA determined that overall disease severity (F(1, 43) = 8.75, *p* < 0.003) and objective fall parameters significantly improved in both mild and severe PD post-intervention (Table 2). However, self-perceived fall risk significantly improved only in the severe PD group F(1, 43) = 9.86, *p* < 0.022) (Figure 1). While no correlations between actual falls and self-perceived fall risk were identified in the mild group (R^2^ = 0.022, *n* = 44, *p* = 0.059), there was a moderate negative correlation in the severe group (R^2^ = −0.289, *n* = 44, *p* = 0.047).

## 4. Discussion

The purpose of the present study was to investigate the influence of an exercise intervention (PD SAFE × TM) on gait characteristics closely linked to falls and the relationship between these characteristics and self-perceived fall risk. Further, to determine the effects of disease severity and balance impairment on this association, participants were stratified into mild PD and severe PD. The current results suggest that PD SAFE × TM improved gait indicators indicative of falls in all participants (both severe and mild PD), but only the severe group showed significant improvements to self-perceived fall risk. 

All gait parameters significantly improved following PD SAFE × TM (stride width (mild = 13.6%; severe = 15.5%), stride width variability (mild = 28.7%; severe = 25.6%)) relatively equally across both mild and severe groups. Notably, certain measures appear to have greater gains in the severe group, although both groups improved significantly, including measures for stride length (mild = 6%; severe = 17.9%), stride length variability (mild = 0.6%; severe = 10.3%), stride time (mild = 58%; severe = 67.9%), and stride time variability (mild = 14.1%; severe = 23.8%)). Additionally, PD SAFE × TM significantly improved overall disease severity in both mild (28%) and severe (31%) groups. Thus, the findings indicate PD SAFE × TM is effective in significantly improving the gait characteristics closely linked to fall risk in the PD population as well as disease severity.

Importantly, the focus of this study was to evaluate whether self-perception of fall risk improved in a similar fashion to objective measures. This is an important consideration as previous studies have noted a possible disconnect between self-perception and objective measures of fall risk in physical rehabilitation [38]. In fact, some argue that the patient’s QOL may be more important than a health professional’s assessment of clinical symptoms, using the UPDRS-III [39]. The current results uncovered that only the severe group improved self-perceived fall risk post-intervention despite both groups improving on clinical assessments. These findings are in agreement with a previous study which found that those with varying disease severities have different relationships between self-perceived and actual balance [40]. This may have important implications for the types of riskier behaviors that people living with PD might engage in. In addition, improvements to self-perceived fall-risk have also been linked to an overall lower risk of falls. A systematic review exploring balance impairments as a risk factor for falls in older adults found that self-reported balance impairments were associated with increased fall-risk, while clinical assessments alone were not [41]. As improved self-perceived fall risk is associated with lower fall risk in general [42], it can be assumed that those in the severe group experienced greater improvement to overall fall risk. 

The improvements seen in self-perceived fall risk in the severe group may also be the result of the progressive approach of PD SAFE × TM, where individuals are continuously challenged. The continual progression through the program may have improved their overall self-perceived fall risk by improving their confidence in their ability to perform ADLs based on improvements seen throughout PD SAFE × TM. Though this study did not monitor ADL and QOL after the intervention directly, previous studies indicate it is also possible that the objective benefits gained from the exercise program may encourage those with severe PD to increase their ADLs outside of the exercise program [40,43]. Together, the improvements to confidence as well as objective measures may result in improved ADLs and overall QOL [39,40,42]. Moreover, improvements to confidence, in general, may lead to fewer falls despite changes to ADL [44]. Another explanation for the improvements seen in this study is that the severe PD group had more room for improvement in both fall risk and disease severity measures [45], meaning that while these objective measures improved, participants were able to notice these improvements and thereby increase their confidence.

## 5. Conclusions

In conclusion, the present study found that PD SAFE × TM improved gait characteristics closely linked to falls across both mild and severe PD groups. However, only the severe group showed improvements to self-perceived fall risk. This was an important finding as self-perceived fall risk has a direct influence on ADL and thereby the QOL of those with Parkinson’s. Thus, self-perceived fall risk may be just as important, if not more important to focus on in the rehabilitation of PD, to improve QOL. This may be an important consideration for future studies and clinicians aiming to investigate fall risk in those suffering from PD as well as older adults in general. The results from this study add to previous work showing that any rehabilitative therapy aiming to improve fall risk should incorporate the evaluation of self-perception as well.

## Figures and Tables

**Table 1:** Demographic characteristics.

	Mild PD (UPDRS-III <20)	Severe PD (UPDRS-III >27)
*N*	22	22
Age (years)	68.12 (8.6)	77.27 (6.52)
No. of years since diagnosis	6.9 (5.0)	8.65 (3.33)
Program adherence %	97.81 (2.25)	98.99 (1.60)
Levodopa equivalent dose (mg/d)	596.45 (320.75)	647.67 (282.63)
Motor severity (UPDRS-III)	13.68 (6.1)	36.66 (8.99)
BMI	27.76 (10.1)	27.59 (6.29)

Abbreviations. UPDRS-III, Unified Parkinson’s Disease Rating Scale motor subsection III.

**Table 2:** Gait parameters closely linked to fall-risk in Parkinson’s disease (PD) before and after a 12-week PD SAFE × TM intervention (standard deviations in parentheses).

	Pre	Post	Effect
Stride width (cm)			
Mild PD	7.41 (5.8)	6.40 (6.1)	Time F(1, 22) = 7.17, *p* < 0.001
Severe PD	12.03 (8.1)	10.17 (7.9)	
Stride width variability (CV)			
Mild PD	7.29 (5.6)	5.20 (6.3)	Time F(1, 22) = 6.56, *p* < 0.017
Severe PD	8.33 (7.0)	6.20 (5.9)	
Stride length (cm)			
Mild PD	53.12 (14.2)	56.36 (12.3)	Time F(1, 22) = 9.37, *p* < 0.0001
Severe PD	48.07 (18.6)	56.67 (15.1)	
Stride length variability (CV)			
Mild PD	8.93 (5.9)	6.19 (5.7)	Time F(1, 22) = 3.87, *p* < 0.039
Severe PD	10.68 (5.6)	6.15 (5.8)	
Stride time (s)			
Mild PD	1.62 (0.59)	0.68 (0.46)	Time F(1, 22) = 9.22, *p* < 0.0001
Severe PD	2.74 (0.60)	0.88 (0.59)	
Stride time variability (CV)			
Mild PD	5.89 (6.3)	5.06 (4.29)	Time F(1, 22) = 3.48, *p* < 0.006
Severe PD	6.72 (8.3)	5.12 (3.19)	

## Data Availability

The data presented in this study are available on request from the corresponding author. The data are not publicly available due to ethical restrictions.
